# Artificial Intelligence Moves Health Care From Reactive Risk Transfer to a Proactive Risk Avoidance System

**DOI:** 10.1016/j.focus.2025.100411

**Published:** 2025-08-07

**Authors:** Duane F. Wisk, Stefan Gravenstein, Denis P.H. Mihale, Manijeh Berenji

**Affiliations:** 1 The Warren Alpert Medical School, Brown University, Providence, Rhode Island; 2 School of Public Health, Brown University, Providence, Rhode Island; 3 Chelsea Management Group, LLC, Jacksonville, Florida; 4 Department of Environmental and Occupational Health, Joe C. Wen School of Population & Public Health, University of California Irvine, Irvine, California

## Abstract

**Figure:**
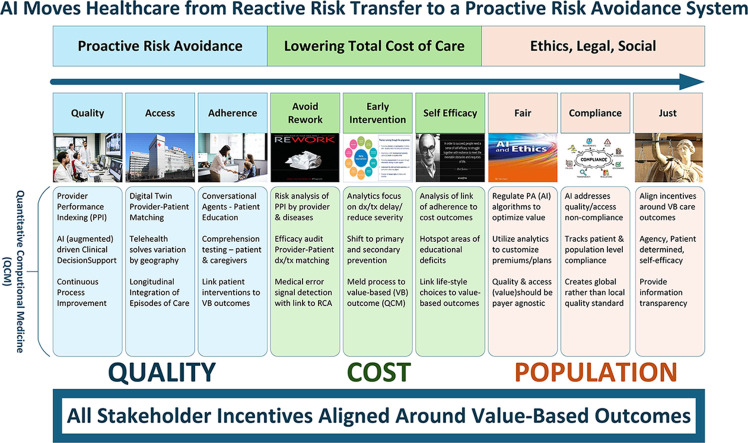

## INTRODUCTION

**Figure 1:**
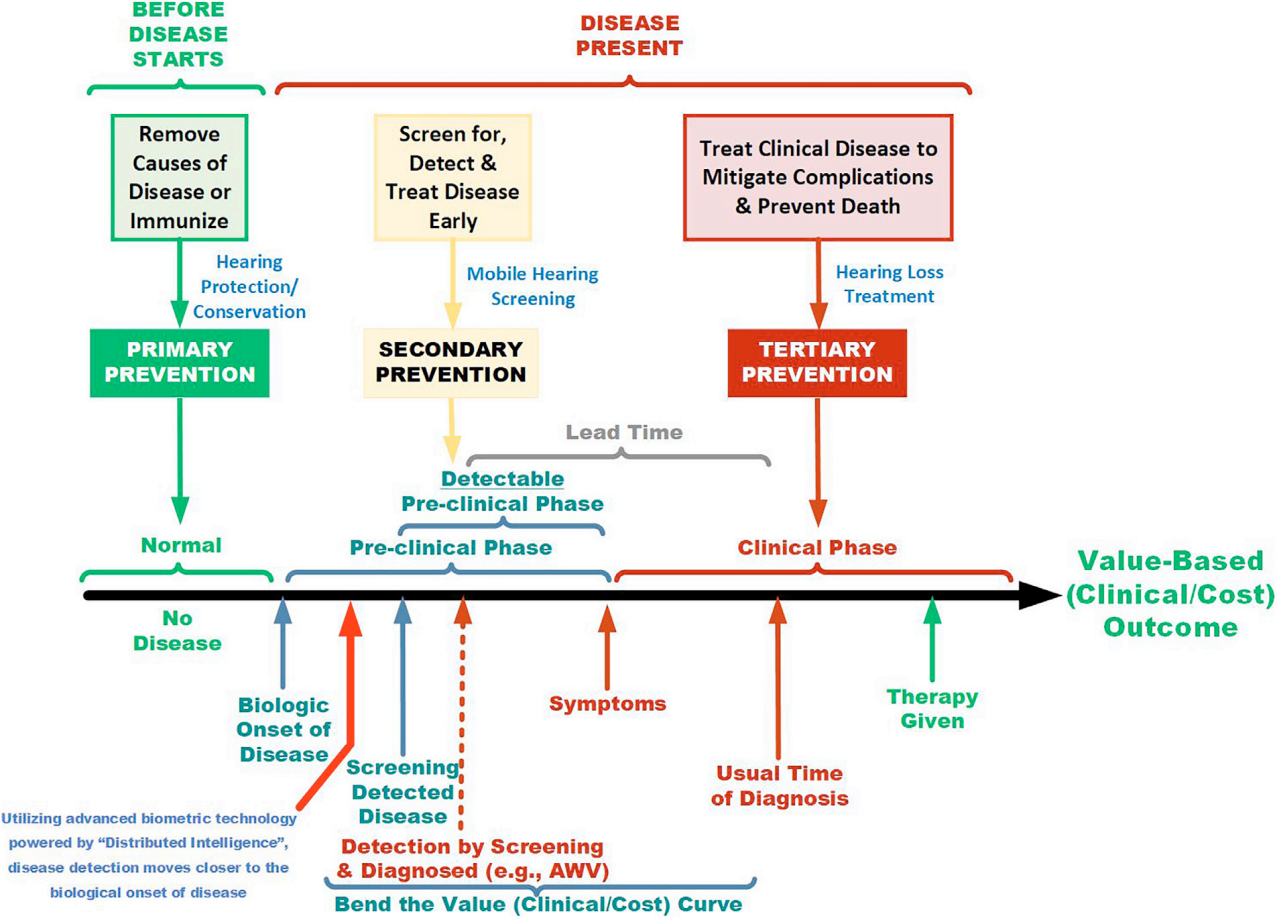
The 3 phases of prevention. The source is Gordis Epidemiology, 6^th^ edition, David D. Celentano and Moyses Szklo, Copyright 2019 by Elsevier, Inc. All rights are reserved. Figure was adapted and modified by the author with permission of Elsevier B.V. through PLSclear.

The purpose of this discussion is to make the case for why artificial intelligence (AI), big data, and precision medicine will shape the future of preventive medicine, public health, and population health, which in turn will shape the future of global healthcare.^1^ This has already been experienced across the healthcare marketplace. Technology enables the evolution of the 2-sided eHealth dynamics of a pull economic model transitioning from a patient-centric (paternalistic) dynamic toward a patient-driven (self-efficacy) focus.^2^ Furthermore, the quantitative computational high-frequency trading technology from finance readies us for its emulation in health care. It makes both Clayton Christensen's^3^ prediction of disruptive healthcare innovation and Michael Porter’s^4^ prediction of a value-based healthcare marketplace possible. The focus of this disruptive innovation should be to shift care from almost exclusively a reactive, tertiary prevention focus to a more balanced allocation of resources between primary and secondary prevention (Figure 1). In doing so, the U.S. healthcare system spends more resources on proactive risk avoidance. However, to get there, a fundamental societal commitment such as the Moon Shot is needed to catalyze and drive transformative cultural change.

## THE KEY CHANGES REQUIRED FOR ARTIFICIAL INTELLIGENCE TO CONVERT THE SYSTEM TO PROACTIVE RISK AVOIDANCE

AI technology can be utilized to enable the healthcare system to shift toward risk avoidance, provided that current obstacles are mitigated or removed altogether. What obstructs the paradigm shift? The healthcare system’s structure, grounded in reactive acute care thinking, consists of nonintegrated systems and stakeholders, outdated legacy systems, and legacy targets.^5^ The system rewards reactive tertiary prevention, which escalates costs. Reactive systems reimburse care for diseases; the emergence of healthcare insurance, first as a benefit to attract employees to jobs and later for protection after retirement, with the introduction of Medicare, fueled this approach. The treatment of disease can more easily be accounted for (and counted) than preventing one could; the former focuses on events in a single person, whereas the latter focuses on a population.^6^ Thus, although primary and secondary prevention reduce overall long-term system costs (you do not pay for what you prevent), monetization remains challenging. Reacting to a problem once it exists runs counter to the intuition of other industry sectors, where improvement efforts, such as Lean, Six Sigma, constraints management, and change management, shape the work.^7^, ^8^ However, all these efforts require data to inform performance and whether process adjustments affect outcomes, that is, one cannot fix what is not measured. With all the digitization since the Health Information Technology for Economic and Clinical Health Act (2009) and the Patient Protection and Affordable Care Act(2010), the U.S. healthcare system still lacks significant systematic approaches to standardization, interoperability, and the efficient and effective measurement of individual patient and population health services.

Therefore, there exist many multifaceted obstacles to advancing population health improvements. Although noteworthy ethical, legal, policy, regulatory, social, and environmental barriers exist, the primary challenge remains economic, not technological or social. The use of technology in the financial sector demonstrates a mastery of nanosecond global asset risk assessment and AI-driven trading technology, culminating in the creation of quant funds that automatically manage trades. Such technology can be applied to enhance disease surveillance; personalize screening, diagnostics, and prognostics; tailor treatments through precision medicine; and inform public health initiatives.^9^, ^10^, ^11^, ^12^, ^13^, ^14^, ^15^ Although not difficult technologically, the path to monetization has eluded us. Demystifying the path requires aligning the incentives of multiple stakeholders within a complex system. Once stakeholders' incentives are aligned, they will drive a collective monetary contribution that gradually converts the healthcare system toward one that focuses more on proactive primary and secondary prevention rather than the reactive one that exists currently. This translates into investments in technology to advance measurement and process improvement prevention efforts.

Each stakeholder must make short-term investments that ultimately accrue long-term savings. In today’s commodity-based healthcare marketplace, cost shifting is a form of risk transfer. When viewed as a whole, risk transfer fuels cost escalation, because competitors do not compete on the value of their goods and services but rather on volume and market share. The U.S. economy, excluding health care, outcompetes those of other countries by improving the value of the goods and services produced by leveraging technology to enhance net productivity (still at 2% per year).^16^ In health care, the quantitative metrics of care value (quantitative medicine) in combination with big data tools are still not measured.^17^ As a result, a value-based marketplace is not operated. If done, competitors would invest more resources into technologies that enhance the value of care outcomes. They will drive technology that improves disease prevention and mitigates early disease progression, enhancing the net long-term value for both the individual and the population. Again, we pay the lower cost of prevention, so we will not have to pay the higher price for what we prevented.

Society must shift its emphasis from reactive tertiary prevention to primary and secondary prevention or proactive risk avoidance. This is a massive process improvement effort. Process improvement begins with measurement, which the U.S. healthcare system does not typically cover.^18^ All stakeholders must invest in measuring the right metrics, not just blind digitization. This means that healthcare insurers must pay for measurement. We must tie that measurement to process improvement that optimizes care outcomes, that is, value-based outcomes for providers and suppliers. Again, the central thesis of Porter’s Redefining Health^4^ is that competitors will shift service and product delivery around value by measuring, analyzing, calculating, and then paying for the value of a healthcare service or product. Now, all stakeholders’ incentives are aligned around a single focus: quantitative computational medicine that precisely calculates and pays for this value-added service or product. Consumers pull demand (2-sided eHealth), shifting away from the field of dreams push economic model. Competition then shifts from a commodity-based to a value-based approach. Interventions for these dynamic, organic, and longitudinal individual and population outcomes are evaluated and incentivized to assess their comparative effectiveness. Stakeholders (e.g., insurers, government, patients, and tech platforms) were not convinced to adopt a value-based approach codified in law by the Affordable Care Act 15 years ago, because no entity has been able to computationally quantify how to measure or calculate the value of a healthcare service or product. Marcel Proust once wrote, “to kindness, to knowledge, we make promise only; pain we obey.” Pain in health care is the remuneration system. If the U.S. healthcare system is to shift to a value-based system (not treatment volume) of proactive risk avoidance that is a consequence of primary and secondary prevention, it must pay for it. To pay for it, the infrastructure to measure, analyze, and compute the quantitative value of that service must first be built. Our only system is based on payment for treatment, where incentives for revenue, driven by volume, influence provider behavior. The service process must be tied to a quantitative outcome (e.g., morbidity, mortality, quality-adjusted life year, disability-adjusted life year, and others) for a complete and accurate picture of its value. These mathematical and technological AI-based tools exist; now they must be utilized. Value-based purchasing will follow, with all stakeholders aligned to value-based incentives.

## ARTIFICIAL INTELLIGENCE ADOPTION AND LOWERING TOTAL COST OF CARE

AI in health care has been shown to quantifiably lower costs in multiple domains, including administrative operations, clinical care, diagnostics, and patient management. Numerous studies and industry analyses provide concrete figures and examples of these savings. The broad adoption of AI could reduce U.S. healthcare spending by 5%–10%, translating into $200–$360 billion annually (in 2019 dollars).^19^ AI can automate administrative tasks such as billing, scheduling, prior authorization, and claims processing. This translates into $150 billion annually. Grouping clinical tasks for AI processing (e.g., using large language models, documentation, cohort selection, and medication reviews) can reduce the costs of running these AI systems by up to 17-fold, resulting in millions of dollars in annual savings for large health systems.^20^ AI-driven diagnostic tools reduce misdiagnoses and unnecessary testing, lowering direct and downstream costs. For example, AI-enabled image analysis in dermatology and cardiology has led to annual savings in specific startups. AI-powered predictive analytics identify high-risk patients, enable early interventions, and reduce hospital readmissions by combining conversational agents with nurse avatar technology (project RED).^21^ AI can accelerate drug discovery and streamline clinical trials, reducing costs and time to market by up to 20%.^22^ Automation of coding and claims reduces human error, recovers lost revenue, and minimizes costly mistakes. AI reduces costs and increases return on investment for healthcare organizations by improving efficiency, accuracy, and patient outcomes.^22^ Over 10 years, AI-based diagnosis and treatment tools can generate exponentially increasing savings as adoption scales and systems mature.^22^ Although AI implementation can require significant upfront investment in technology and training, the long-term cost reductions are substantial. Realizing these savings depends on effective integration, regulatory approval, and acceptance by clinicians and administrators. This may be the biggest challenge. To reiterate, AI technology demonstrably lowers healthcare costs in quantifiable terms. Estimates range from $150 billion to $360 billion in annual savings for the U.S. healthcare system, with reductions stemming from administrative automation, improved diagnostics, reduced readmissions, and streamlined operations. These savings are supported by large-scale economic analyses and real-world case studies, which confirm that AI adoption can make health care more affordable without compromising quality or access.^19^

## ETHICAL, LEGAL, AND SOCIAL IMPLICATIONS OF ARTIFICIAL INTELLIGENCE IN CLINICAL PRACTICE

**Figure 2:**
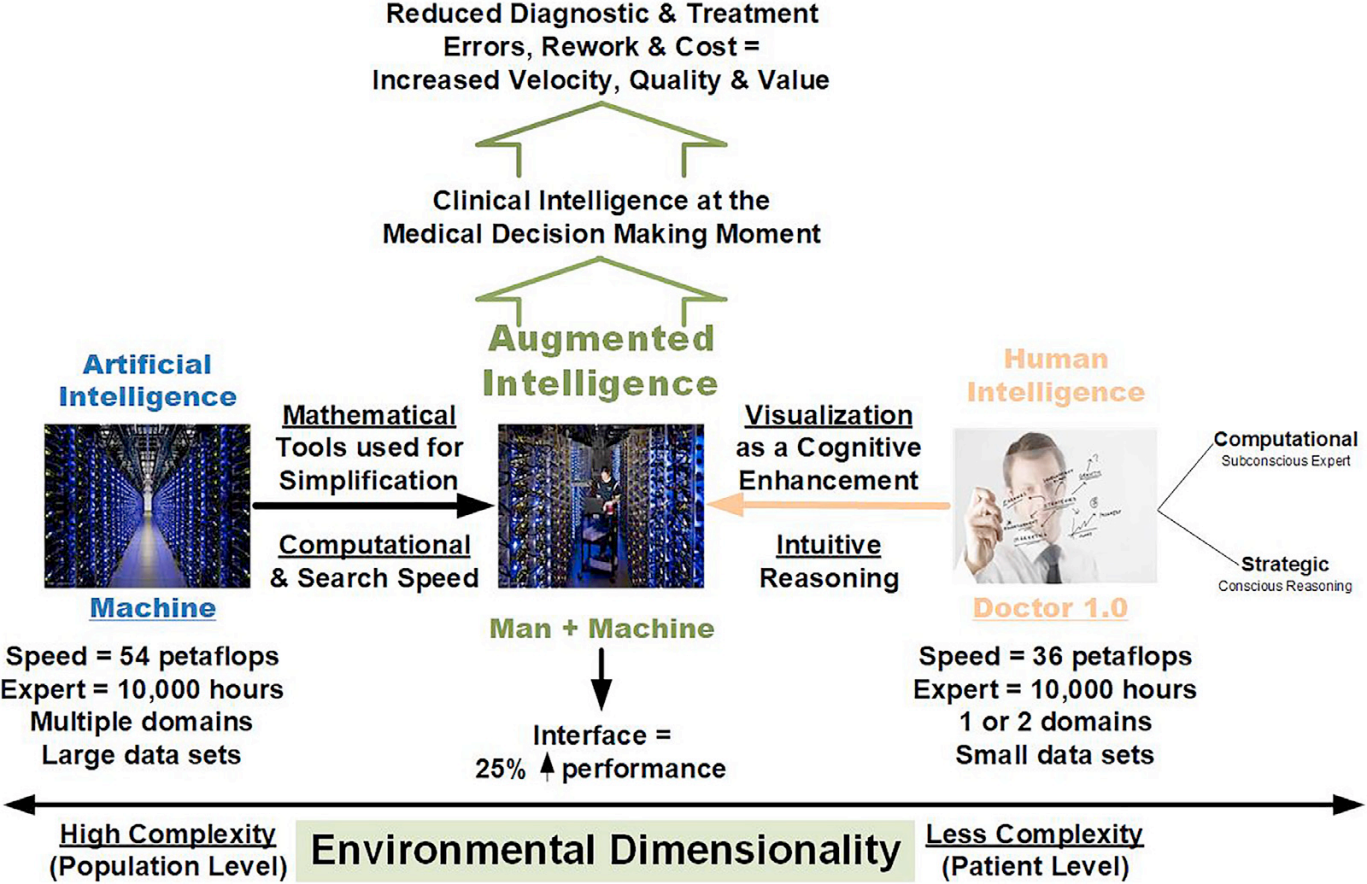
AMI. *Note*: The source is the author. AMI, augmented medical intelligence.

As AI becomes more prevalent in health care, clinicians must navigate a range of ethical, legal, and social considerations. A key concern is bias—many AI systems are built on generalized, qualitative data that can perpetuate existing disparities in care. The approach should be fundamentally different: it should rely exclusively on structured, analytical data drawn from patient history and apply a risk-based framework inspired by the Capital Asset Pricing Model. This ensures that decisions are informed by objective, quantifiable input rather than assumptions or demographic shortcuts. The goal should be to support rather than replace clinical judgment, while augmenting it (Figure 2). By minimizing qualitative bias and grounding recommendations in patient-specific risk analysis, clinicians are provided with tools to make the most informed decisions possible—decisions tailored to each individual, not statistical averages. As clinicians, it is our responsibility to protect patient privacy. AI systems must be designed with strong safeguards that meet and exceed Health Insurance Portability and Accountability Act requirements. The data must be handled securely, with transparency built into the process so that all stakeholders know how and why an output was generated. Finally, clear boundaries of accountability must be maintained. AI recommendations should be both traceable and auditable, and clinical decision making should remain a joint, patient- and clinician-driven process. The contributing factors—from the algorithm’s logic to the source data—can be examined and understood if something goes wrong. Keeping AI grounded in data, risk management, and clinical transparency will make it a reliable ally in patient care, not a black box but a decision-support system that clinicians can trust. With such an ethical proviso, AI can prioritize primary and secondary prevention at the forefront of societal and, consequently, healthcare resources, shifting the system toward a proactive risk avoidance paradigm.

## CONCLUSIONS

AI technology then has a specific placement in the healthcare process and, therefore, care outcomes. This approach moves our post-2009 productivity-crushing electronic health records 1.0 to their replacement by productivity-enhancing electronic health records 2.0. Data from technology will indicate how to match the roles and workflows of each caregiver to the top of their license. An augmented intelligence architecture will optimize disease surveillance; customize screening, diagnostics, and prognostics; and tailor treatment through precision medicine as well as tailor public health initiatives. For example, we can apply digital twin technology (developed by National Aeronautics and Space Administration) at the patient, provider, and population levels. We can utilize conversational agents (natural language processing/natural language generation and other large language model technologies) as workforce multipliers in patient education to enhance the transitions of the care process. We can apply data mesh techniques to enhance data management, improving data creation and sharing.^23^ We use them as a *lingua franca* as the Office of the National Coordinator for Health Information Technology seeks to optimize interoperability between disparate systems and vendors. We can further deploy other AI tools that enhance clinical decision support to improve diagnostic and treatment outcomes. We can use all these existing and undiscovered tools to shape the future of preventive medicine, public health, and population health. As a society, we must decide that all stakeholders' front-loaded investments today in these technologies directed to primary and secondary prevention are the first step to capturing significant savings tomorrow.^24^

## CRediT authorship contribution statement

**Duane F. Wisk:** Conceptualization, Writing – original draft, Writing – review & editing. **Stefan Gravenstein:** Writing – review & editing. **Denis P.H. Mihale:** Writing – review & editing. **Manijeh Berenji:** Writing – review & editing.
